# Saccadic Velocity in the New Suppression Head Impulse Test: A New Indicator of Horizontal Vestibular Canal Paresis and of Vestibular Compensation

**DOI:** 10.3389/fneur.2016.00160

**Published:** 2016-09-23

**Authors:** Qiwen Shen, Christophe Magnani, Olivier Sterkers, Georges Lamas, Pierre-Paul Vidal, Julien Sadoun, Ian S. Curthoys, Catherine de Waele

**Affiliations:** ^1^Cognition and Action Group, CNRS UMR8257, Centre Universitaire des Saints-Peres, Universite Paris Descartes, Paris, France; ^2^ENT Department, Salpetriere Hospital, Paris, France; ^3^Vestibular Research Laboratory, School of Psychology, University of Sydney, Sydney, NSW, Australia

**Keywords:** video-head impulses, horizontal vestibule–ocular reflex, saccade substitution, vestibular loss, bilateral areflexia, IT gentamicin, vestibular schwannoma, Meniere’s disease

## Abstract

**Objective:**

To determine whether saccadic velocity in the suppression head impulse paradigm (SHIMP) test is a reliable indicator of vestibular loss at the acute and at the chronic stage in patients suffering from different vestibular pathologies.

**Methods:**

Thirty-five normal subjects and 57 patients suffering from different vestibular pathologies associated with unilateral vestibular loss (UVL) or bilateral vestibular loss (BVL) were tested in the SHIMPs paradigm. SHIMPs were performed by turning the head 10 times at high velocities to the left or right side, respectively. The patients were instructed to fixate on a red spot generated by a head-fixed laser projected on the wall. In this SHIMPs paradigm, healthy subjects made a large anti-compensatory saccade at the end of the head turn (a SHIMP saccade). The peak saccadic velocity, the percentage of the trials completed with saccades in 10 trials, and the latency of the saccades were quantified in each group. A video-head impulse test (v-HIT) was systematically performed in all of our subjects as well as a caloric test. The dizziness handicap inventory questionnaire was also given to chronic UVL and BVL patients.

**Results:**

At the acute stage after a complete UVL, patients had zero or a few anti-compensatory saccades for low velocity head turns toward the lesioned side. These saccades had lower velocity than the anti-compensatory saccades recorded during head rotation toward the intact side and/or compared with the saccades measured in control subjects. At the chronic stage, some of the patients recovered the ability to perform SHIMP saccades at each head turn toward the lesioned side, but very often these saccades were of significantly lower velocity. In BVL patients, no anti-compensatory saccades, or only significantly smaller ones, could be detected for head turns to both sides.

**Conclusion:**

SHIMP is a specific and sensitive test to detect a complete horizontal canal loss at the acute stage. In addition, it reflects the ability of patients with moderate horizontal vestibulo–ocular reflex gain decrease to generate anti-compensatory saccades in the chronic stage. In association with v-HIT, it allows determination of the residual vestibular function and to detect anti-compensatory saccades.

## Introduction

Video-head impulse test (v-HIT) was recently developed to measure the gain of the vestibulo–ocular reflex (VOR) in the horizontal and the vertical plane for testing the horizontal, anterior, and posterior canals (1–6). This test is now called the head impulse test (HIMP). v-HIT testing also shows how covert and overt catch-up saccades compensate for the deficient VOR. Interestingly, patients suffering from complete unilateral vestibular loss (UVL) often complain about oscillopsia, which persists over time despite fast covert compensatory saccades. Recently, Ian Curthoys’ group developed a new test (7): the suppression Head Impulse Paradigm (SHIMP). In this paradigm, the patient is asked to follow a red spot on the wall generated by a laser secured to his/her head, while the clinician delivers the head impulse. In case of intact vestibular function, the horizontal VOR (HVOR) drives the eyes to the opposite side to the head rotation during the first 80 ms and, therefore, contralateral to the head-fixed target movement. Hence, the subject has to generate a large anti-compensatory saccade (a SHIMP saccade) to reacquire the target at the end of the head turn. Following a vestibular lesion, the HVOR is deficient and, therefore, the slow phase it generates drives the eyes through a smaller distance than the target so that the size of the corrective SHIMP saccades is smaller (7). When the HVOR gain is absent due to a complete lesion, no saccade is required. The eye movement recording in SHIMPs during head impulses also evaluates the VOR gain as it does in the standard HIMPs paradigm.

In this work, we explored the complementarity of SHIMPs and HIMPs in a well-defined cohort of UVL patients and bilateral vestibular loss (BVL) patients at two different stages: acute and chronic. SHIMPs and HIMPs were performed sequentially. The data were compared with the data of a control group of healthy, asymptomatic subjects. Our aim was to identify the extent to which saccadic velocity can be used to index vestibular loss. We also tried to understand the difference between compensatory in HIMPs and anti-compensatory saccades in SHIMPs.

## Materials and Methods

Ninety-two patients, including 35 normal subjects (13 men and 22 women; mean age 54 ± 15; min–max: 20–80) and 57 vestibular patients (34 men and 23 women; mean age 58 ± 13; min–max: 23–87), were tested for their horizontal canal function using both HIMPs and SHIMPs. The normal subjects had no neurological problem or inner ear pathologies. The vestibular patient composed acute UVL and chronic UVL groups. Acute UVL group (mean age 56 ± 15; min–max: 23–87) included 23 patients operated from unilateral vestibular schwannoma tested within 6 weeks after surgery. All the patients tested at the acute stage had a spontaneous ocular nystagmus with the quick phase oriented toward the intact side in the sitting and supine position. Chronic UVL group (mean age 58 ± 10; min–max: 37–74) was composed of 28 patients with vestibular schwannoma operated longer than 6 weeks (*n* = 7), or patients with vestibular schwannoma removed by gamma knife (*n* = 8), or patients suffering from Meniere’s disease and treated by intratympanic gentamycin injections (40 mg/mL) three times with 1-week interval in between (*n* = 13). All these chronic patients were areflexic to the caloric test on the lesioned side. They exhibited a positive head shaking nystagmus and vibratory nystagmus with the quick phase oriented toward the intact side. BVL patients from unknown origin, “BVL” group (mean age 62 ± 18; min–max: 36–83), included six patients with complete bilateral peripheral vestibular deficit on bilateral caloric testing and horizontal v-HIT. They were all at a chronic stage. They were also areflexic to cervical and ocular VEMPs. The etiology cannot be determined exactly despite a lot of blood and radiologic test (MRI, CT-scan) (8–10). All subjects were informed about the vestibular tests, and gave a written-informed consent. The Clinical Research Ethics Committee approved this work, registered at ANSM (ID RCB 2014-A00222-45).

### Video-Head Impulse Paradigm

Horizontal video-HIT (OtosuiteV^®^, GN Otometrics, Denmark) was used to assess the function of the horizontal semicircular canal as previously described (1). Subjects were instructed to fixate an earth-fixed laser dot on the wall at 90 cm distance on the wall in front of the patient. Twenty horizontal head impulses were manually applied to each side with unpredictable timing and direction by the clinician. The amplitude of the head rotation was about 18–20°, and the peak head velocity of the impulse was about 180–220°/s and of acceleration between 4500 and 7500°/s^2^. Eye velocity and head velocity were recorded for each head rotation. The VOR gain was calculated as the ratio of the area under the de-saccaded eye velocity to the area under the head velocity (2, 11).

### Suppression Head Impulse Paradigm

The experiment followed exactly the same procedure that was used for HIMP with one exception. Participants were instructed to fixate a laser spot target projecting on the wall in front of them from a head-mounted laser, which moved with the head (7). Ten impulses were delivered to the left and right side, respectively. Eye velocity and head velocity were recorded in each head rotation.

**Figure 1:**
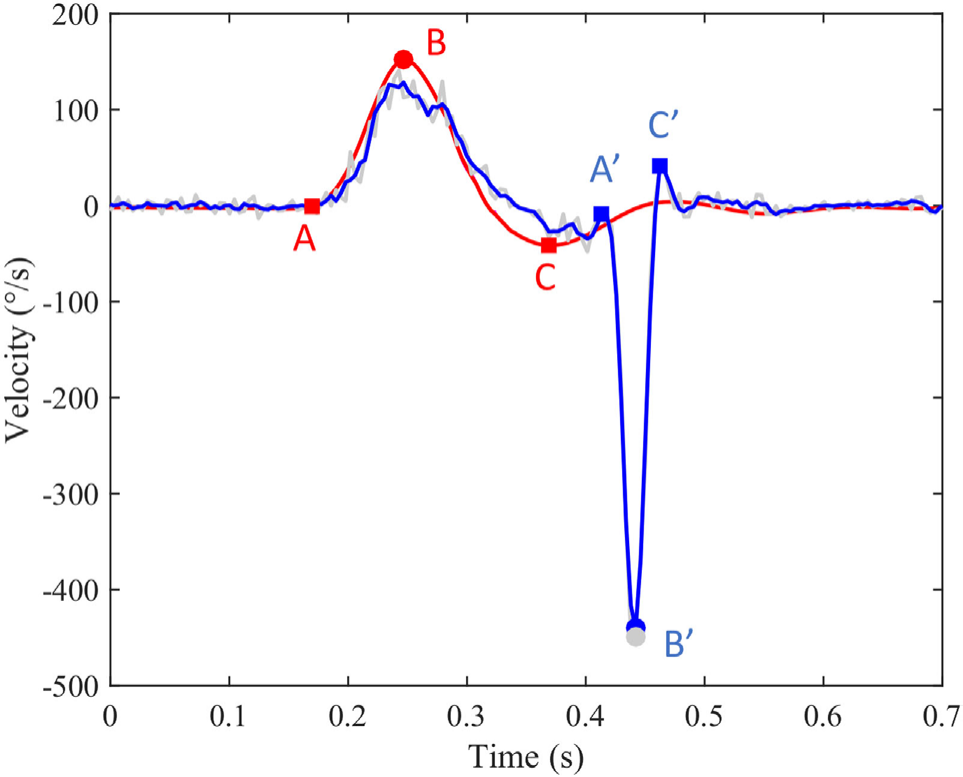
**An example of filtered head velocity superimposed with filtered eye velocity and non-filtered eye velocity**. Red curve: head velocity; blue curve: eye velocity; blue circle: peak saccade velocity; red circle: peak head velocity; gray circles: non-filtered peak saccade velocity.

An algorithm was developed in MATLAB R2016a (The MathWorks, Inc., USA) to process ASCII data files supplied by ICS Impulse (GN Otometrics, Denmark). The raw data contain head velocity and eye velocity in degrees per second. These are noisy signals of quasi-zero mean superimposed with head impulses or saccadic eye reactions. The undesirable noise was reduced with a least-squares smoothing polynomial filter preserving as much as possible the high frequency content of impulses and saccades. Figure 1 shows an example of filtered head velocity (red curve) superimposed with filtered eye velocity (blue curve) and non-filtered eye velocity (gray curve). Although all computations were done with filtered signals, the gray curve is useful to check manually the quality of filtering as well as occasional artifacts caused by the hardware sampling. A head impulse is characterized by three events (A, B, C) over time. The first event (A) marks the beginning of the head impulse when it moves away from 0°/s. The second event (B) marks the peak velocity of the head impulse, which was selected to be at least 50°/s. The sign of the peak velocity indicates the orientation left (+) or right (−) of the head rotation. The third event (C) marks the overshoot, which is followed by a damped oscillation returning to 0. Trials with overshoot more than 50°/s were excluded for further analysis. A saccadic eye reaction can occur (or not occur) after a head impulse with a specific latency defined below. The algorithm implements saccade detection for a minimal velocity (50–200°/s) and a maximum head-peak to eye-peak duration (600 ms). A saccade is characterized by three events (A′, B′, C′) over time. The first event (A′) marks the bifurcation between the slow phase velocity induced by the VOR and the saccadic movement. The second event (B′) marks the peak velocity of the saccade. The third event (C′) marks the end of the saccade before returning to the normal state. The sign of the eye curve is inverted in order to facilitate the comparison with the head curve. A new index, the ratio between peak saccade velocity and peak head velocity was used to access the residual vestibular function in patients. Latency corresponds to the time interval between the onset of the head impulse and the onset of the saccade response. Following the definition of Findlay and Walker (12), the latency used in this paper is mathematically defined as the time interval AA′ from the head start event A to the eye start event A′. Statistical analysis of long duration experiments was done by cutting data into pieces isolating each head impulse with its associated saccadic eye reaction. All pieces were superimposed with head peaks aligned, showing 0.25 s to the left and 0.6 s to the right.

### Caloric Test

Caloric tests were performed using sequential bithermal external ear canal irrigations with water at 30 and 44°C as previously reported (13). The peak velocity of the induced ocular nystagmus was recorded by video-nystagmography (Synapsys, France) on each side with warm and cold stimulation. Percentage of canal paresis (CP) was calculated using Jongkees’ formula: CP = 100 × [(LW + LC) − (RW + RC)]/LW + LC + RW + RC, in which LW, LC, RW, and RC are the maximum velocity of the induced ocular nystagmus obtained on the left (L) and right (R) sides, with warm (W) and cold (C) stimulation. CP value above 25% was defined as an abnormal response.

### Dizziness Handicap Inventory and Oscillopsia Complaint

Dizziness handicap inventory (DHI) questionnaire is a self-assessment inventory including 25 questions to evaluate self-perceived activity limitation and restriction resulting from dizziness (14). It was given to all normal subjects, each UVL patient at chronic stage, and all BVL patients. All UVL patients at acute stage were asked whether they had oscillopsia during rapid horizontal head turn in their daily life.

### Statistical Analysis

The mean peak saccade velocity, mean percentage of saccade responses in SHIMPs, and HIMP VOR gain and mean peak head velocity for the trials of each patient were calculated as the sum for each side from 10 trials divided by the number of trials. When no saccade was detected in a particular trial, the peak saccade velocity was considered as zero. The significant difference of peak saccade velocity and saccade latency was calculated by paired sample *t*-test (significance level *p* < 0.05). The receiver operating characteristic (ROC) statistics were calculated with XLSTATS (New York, USA).

## Results

### Preliminary Saccade Analysis

The anti-compensatory saccade obtained in SHIMPs in this study followed the saccade main sequence characteristics (15, 16).

### The Normal Subjects with Normal v-HIT

**Figure 2:**
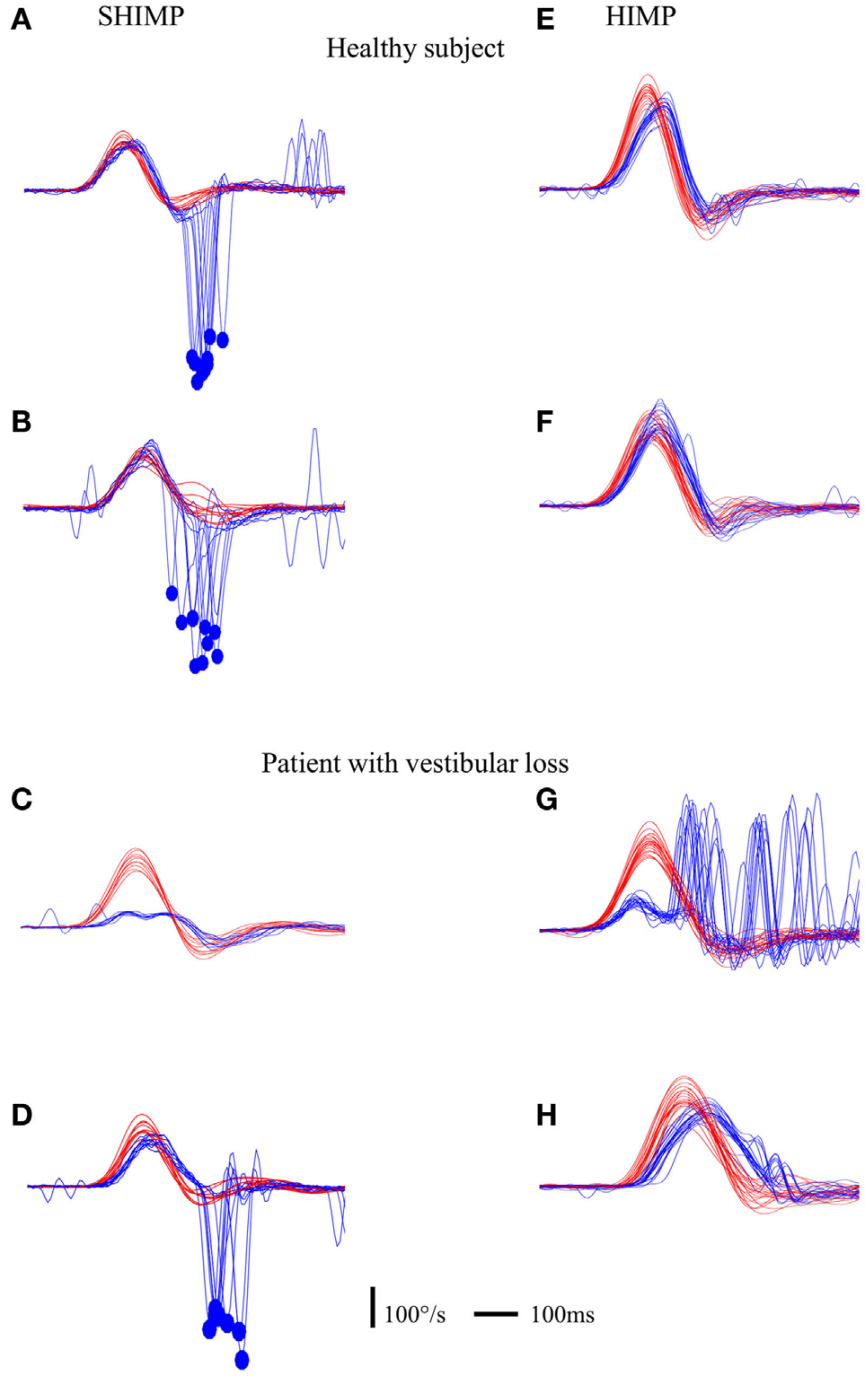
**Illustration of compensatory and anti-compensatory saccades obtained in SHIMP and HIMP procedures, respectively**. **(A–D)** SHIMP raw data in a normal subject when the head impulse was directed toward the left **(A)** and right **(B)** side and in a complete UVL patient when the head impulse was directed toward the right lesioned **(C)** and left intact **(D)** side. **(E,F)** HIMP raw data in a normal subject when the head impulse was directed toward the left **(E)** and right **(F)** side and in a complete UVL patient when the head impulse was directed toward the right lesioned **(G)** and left intact **(H)** side. Red curve: head velocity; blue curve: eye velocity; blue circle: peak saccade velocity; vertical bar: 100°/s; horizontal bar: 100 ms.

**Figure 3:**
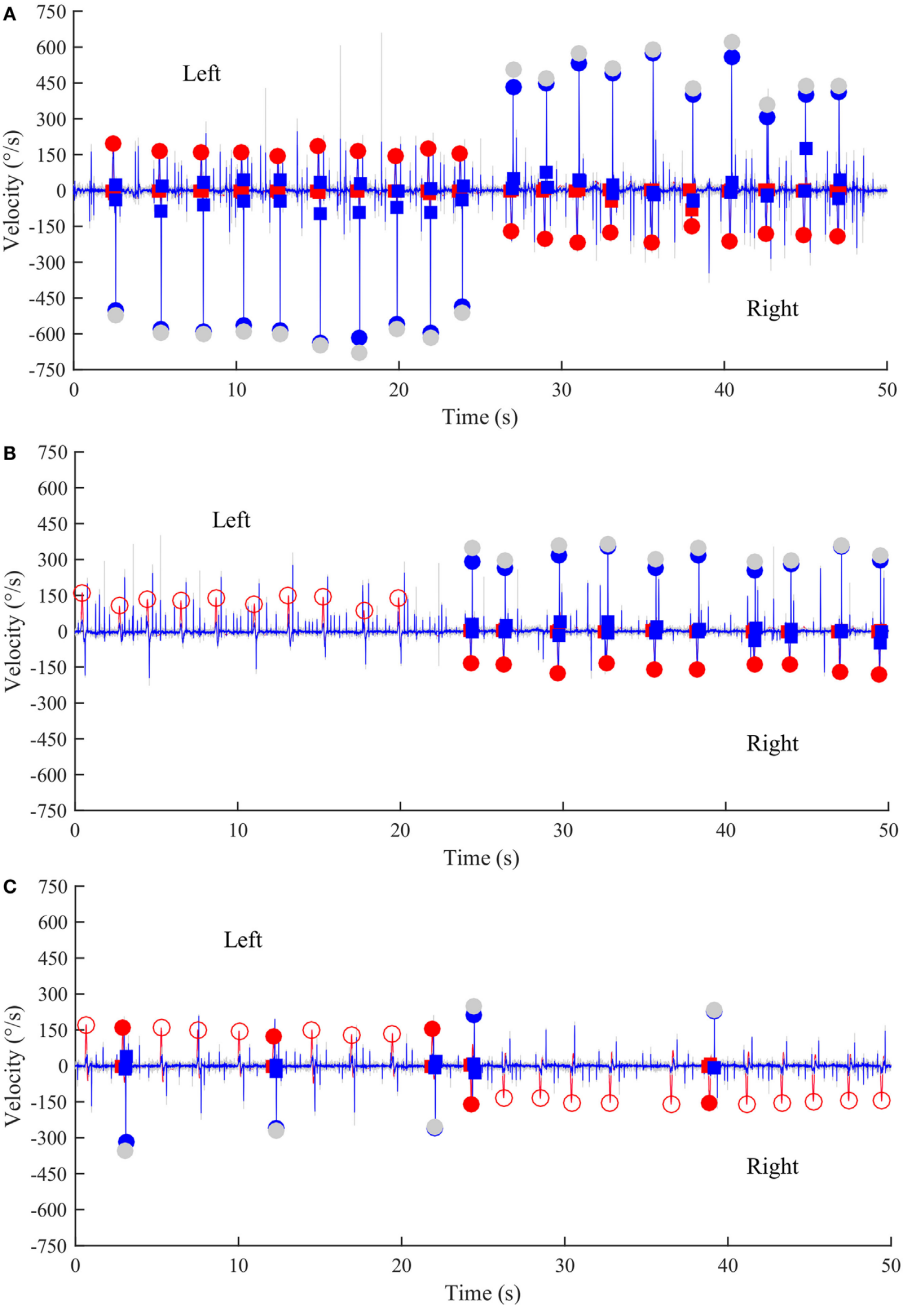
**Time series of anti-compensatory saccades in a normal subject, a left UVL patient, and a BVL patient**. **(A)** Anti-compensatory saccades were detected when head impulses were directed either to the left (up, 2–24 s) or to the right side (down, from 27–47 s) in a normal subject. **(B)** No anti-compensatory saccades were detected when head impulses were directed toward the lesioned left side (up, 0–20 s) in a patient operated from a left vestibular schwannoma and tested in acute stage. In contrast, anti-compensatory saccades could be detected for head impulses toward the intact right side (down, 24–50 s). **(C)** Few anti-compensatory saccades were detected when head impulses were directed either toward the left (up, 0–22 s) or toward the right side (down, 24–50 s) in a BVL patient. Red curve: head velocity; blue curve: eye velocity; blue circles: peak saccade velocity; red circles: peak head velocity; empty red circles: head impulses in which anti-compensatory saccades were not followed after the head turn; gray circles: non-filtered peak saccade velocity.

**Figure 4:**
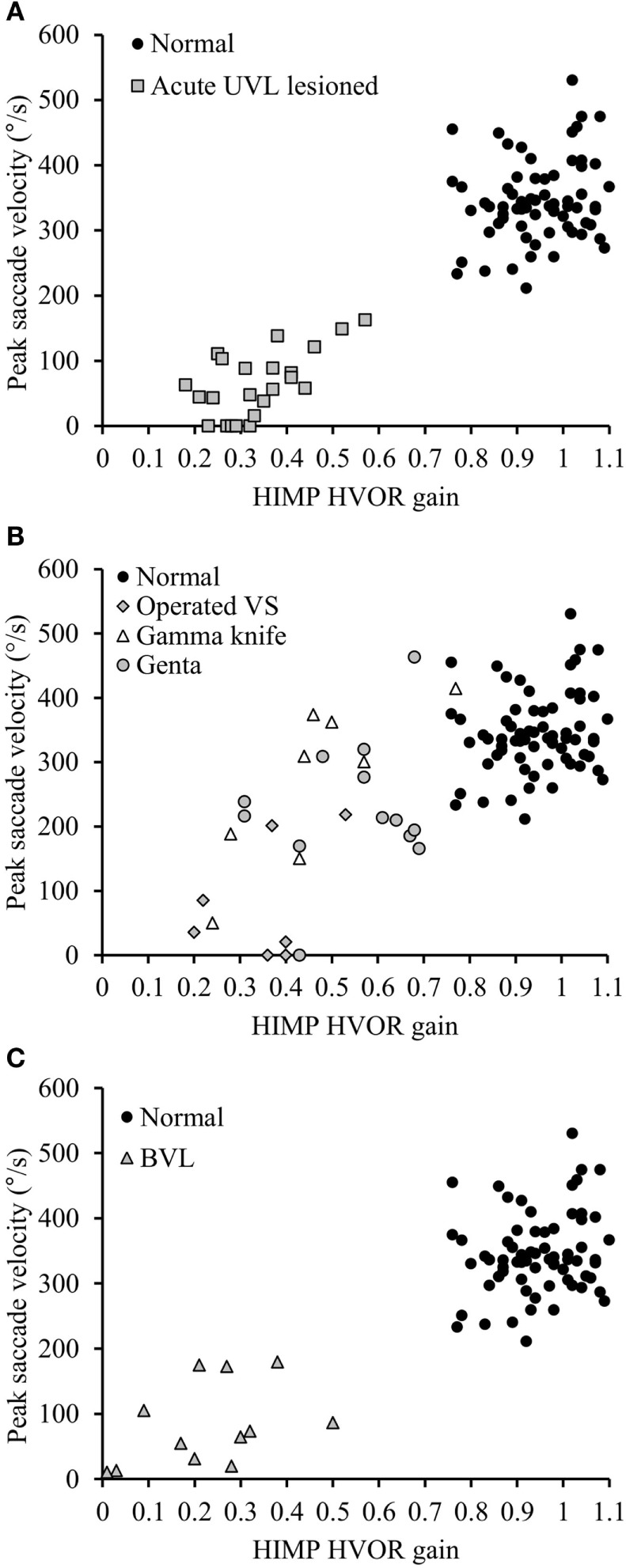
**Peak saccade velocity in function HIMP HVOR gain in normal subjects and in patients suffering from different vestibular pathologies and tested at different stages following the lesion**. **(A)** Peak saccade velocity in UVL patients operated for vestibular schwannoma tested at acute stage and normal subjects. Notice that when HIMP gain is low, the peak saccade velocity in acute UVL patients (gray squares) was significantly lower than the ones in normal subjects (black circles). **(B)** Patients suffering from different vestibular pathologies and areflexic to the caloric test were tested with SHIMPs at chronic stage. Again that the SHIMPs peak saccade velocity vary in function of the HIMP HVOR gain. Gray diamonds: patients with vestibular schwannoma operated after 6 weeks; empty triangle: patients with vestibular schwannoma treated by gamma knife; gray circles: patients suffering from Meniere’s disease and treated by intratympanic gentamycin injection. **(C)** Peak saccade velocity in BVL Patients and normal subjects. Notice that when HIMP gain is low, the peak saccade velocity in BVL patients (gray triangles) was significantly lower than the ones in normal subjects (black circles).

**Figure 5:**
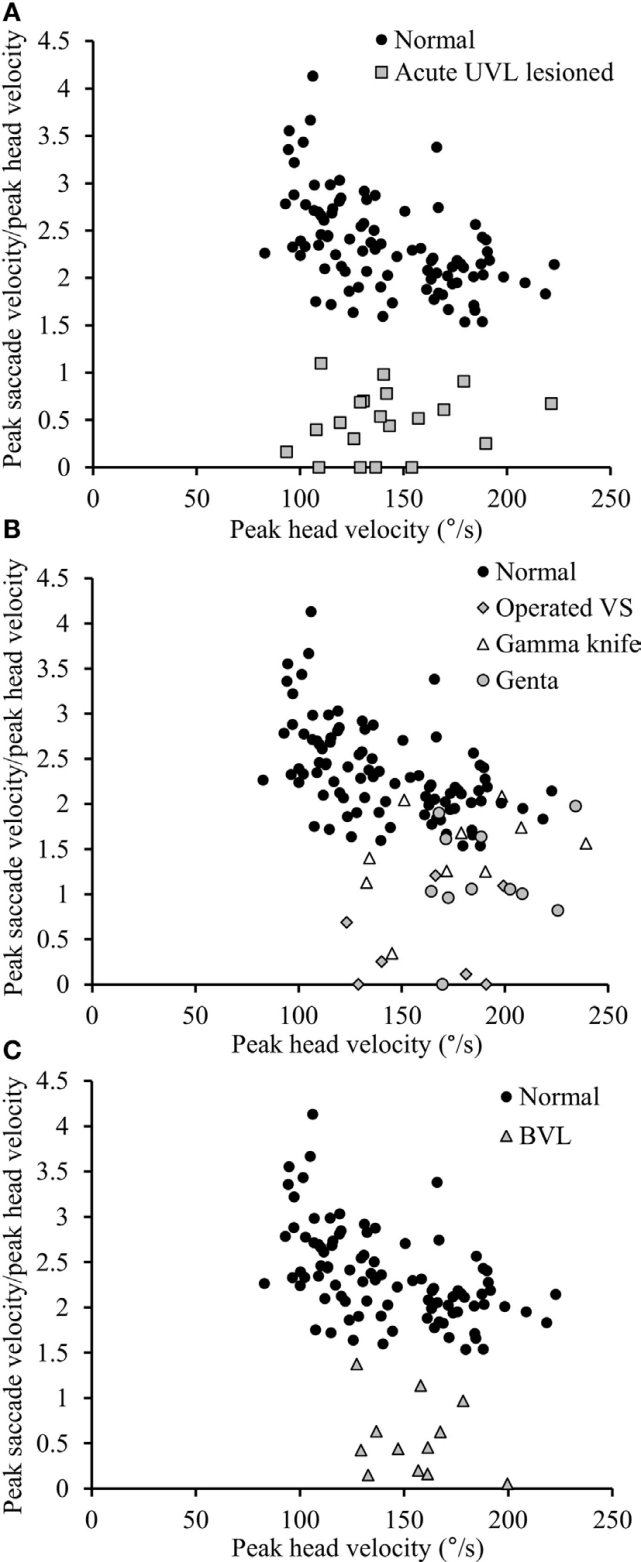
**Relationship between peak saccade velocity and peak head velocity**. **(A)** The ratio between peak saccade velocity (°/s) and peak head velocity (°/s) is close to 2.5 in normal subjects (black circles), whereas it is lower than 1.10 in acute UVL patients (gray squares). **(B)** The ratio varies from 0 to normal values in chronic UVL patients. Notice that normal values were only seen in some patients treated with gamma-knife therapy because of unilateral vestibular schwannoma (empty triangle) or patients suffering from Meniere’s disease and treated by intratympanic gentamycin injections (gray circles), but not patients with vestibular schwannoma operated after 6 weeks (gray diamonds). **(C)** The ratio in BVL patients (gray triangles) is lower than 1.37.

**Figure 6:**
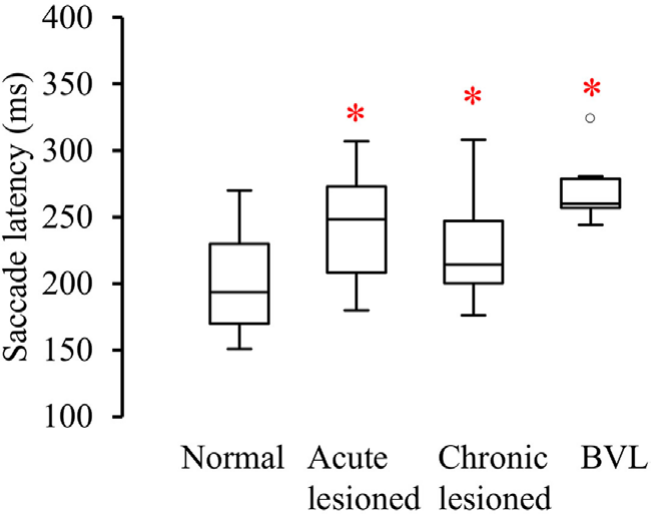
**Latency between the start of head turn and the start of the anti-compensatory saccade (ms) in normal subjects, acute UVL, chronic UVL, and BVL patients**. The bottom and the top of the box represent the 25th and 75th percentiles, and the band within the box represents the 50th percentile. The ends of the whiskers represent the maximum and minimum of all the data. Asterisks indicate significantly increased saccade latency in the lesioned side of acute UVL patients (*p* < 0.005), the lesioned side of chronic UVL patients (*p* < 0.02), and BVL patients (*p* < 0.0001).

Healthy controls performed large anti-compensatory saccades immediately after head turn in SHIMPs (Figure 2A). When the head impulse was delivered to the left side, the eyes moved toward the right, and, at the end of the head impulse, the subject had to make a reflexive saccade to the left direction to regain fixation of the target. When the head impulse was delivered to the right side (Figure 2B), the eyes moved toward the left because of the slow phase of HVOR, and the subjects had to make a reflexive saccade to the right to regain fixation of the target (Video S2 in Supplementary Material). In contrast, healthy controls completed HIMPs without any saccades on both left and right sides (Figures 2E,F). In healthy subjects, SHIMPs saccades were detected for every head turn on both sides (i.e., 10/10 trials) (Figure 3A). No significant difference was found between the left and right sides of normal subjects below 65 years, or between left and right sides of senior normal subjects, or between normal subjects below 65 years and senior normal subjects (Table 1). Taking into account all normal subjects, the mean peak saccade velocity of the left and right sides in controls was 347 ± 66°/s and 346 ± 61°/s (Table 1). Healthy subjects showed significantly higher peak saccade velocity (mean: 354 ± 63°/s; min–max: 21–530°/s) and higher HIMPs VOR gain (mean: 0.96 ± 0.11; min–max: 0.76–0.10) compared with vestibular patients (Figure 4A). The ratio between peak saccade velocity and peak head velocity was always close to 2.5 (Figure 5A). The mean latency of the saccades of healthy subjects was 201 ± 32 ms (min–max: 151–270 ms) (Figure 6).

**Table 1:** **Mean peak saccade velocity (°/s) in normal subjects (>65 years), normal subjects (<65 years), BVL patients, acute UVL patients, and chronic UVL patients**.

Peak saccade velocity (°/s)		
	**Left side**	**Right side**
Normal (<65 years)	342 ± 48	343 ± 60
Normal (≥65 years)	358 ± 101	346 ± 65
BVL	104 ± 81*	60 ± 35*
	**Lesioned side**	**Intact side**
Acute UVL	64 ± 50^#^	354 ± 77
Chronic UVL	202 ± 129^#^	329 ± 86

Notice the significant decrease of peak saccade velocity in BVL patients on both sides compared with that in normal subjects (* indicates significant difference in *t*-test, *p* < 0.05). The peak saccade velocity in acute UVL and chronic UVL patients were also significantly smaller compared with their corresponding sides (^#^ indicates significant difference in *t*-test, *p* < 0.05).

### The Vestibular Patients

We studied three groups of vestibular patients (*n* = 57) in this study: acute UVL patients, chronic UVL patients, and BVL patients. All vestibular patients included in this study were areflexic to caloric test and had 100% CP.

#### Acute UVL Patients

Acute UVL patients (*n* = 23) elicited no or a few SHIMP saccades when the head was turned to the lesioned side (Figure 2C), whereas they exhibited large SHIMP saccades for head turn in the intact side (Figure 2D and Figure 3B). When doing HIMPs, acute UVL patients elicited large compensatory saccades when the head was turned to the lesioned side (Figure 2G). However, they completed head turn without saccades in the intact side (Figure 2H). Acute UVL patients showed significantly lower saccade velocity in SHIMPs compared with healthy subjects (mean: 64 ± 50; min–max: 0–163°/s) and also had decreased VOR gain in HIMPs (mean: 0.34 ± 0.10; min–max: 0.18–0.57) to their lesioned side (Figure 4A). Also, the ratio of the saccade velocity to peak head velocity in the lesioned side of acute UVL group was from 0 to 1.10, which was much smaller compared with that in normal subjects (mean of 2.5) (Figure 5A). The mean peak saccade velocity for head turns to the lesioned side of acute UVL patients was 64 ± 50°/s. It was significantly lower compared with that toward the intact side (354 ± 77°/s). With the analysis of all acute UVL patients, not only the mean peak saccade velocity but also the percentage of saccadic response in SHIMPs and the VOR gain in HIMPs were significantly lower in the lesioned side of acute UVL patients compared with those in their intact side. Acute UVL patients were able to perform saccadic response 100% in SHIMPs on their intact side. However, in average, only 34% of the head impulses on their lesioned sides were completed with saccadic response (Table 2). Turns to the lesioned side of acute UVL patients produced saccades with a mean latency of 241 ± 40 ms, which were significantly longer compared with that in normal subjects (201 ± 32 ms) (*p* < 0.005) (Figure 6).

**Table 2:** **Peak saccade velocity, percentage of response in SHIMPs, and HIMP VOR gain in the lesioned and intact side of acute UVL patients**.

	Lesioned side				Intact side		
	Stage (weeks)	Peak saccade velocity (°/s)	% of response in SHIMP	HVOR gain HIMP	Peak saccade velocity (°/s)	% of response in SHIMP	HVOR gainHIMP
Patient 1	1	89	57	0.37	391	100	0.84
Patient 2	1	38	33	0.35	519	100	0.79
Patient 3	1	81	48	0.41	438	100	0.80
Patient 4	1	74	57	0.41	412	100	0.96
Patient 5	1	15	15	0.33	251	100	0.74
Patient 6	1	58	10	0.44	324	100	0.79
Patient 7	1	88	30	0.31	311	100	0.73
Patient 8	1	111	55	0.25	370	100	0.91
Patient 9	1	0	0	0.27	242	100	1.11
Patient 10	1	121	62	0.46	406	100	0.98
Patient 11	1	56	67	0.37	495	100	0.85
Patient 12	1	138	58	0.38	335	100	0.98
Patient 13	1	43	20	0.24	335	100	0.82
Patient 14	1	0	0	0.29	341	100	0.99
Patient 15	3	0	0	0.28	342	100	0.83
Patient 16	3	149	46	0.52	376	100	0.74
Patient 17	3	103	55	0.26	210	100	1.02
Patient 18	3	63	25	0.18	268	100	0.84
Patient 19	3	44	20	0.21	335	100	0.73
Patient 20	6	0	0	0.23	285	100	0.78
Patient 21	6	163	75	0.57	342	100	0.78
Patient 22	6	0	0	0.32	437	100	0.76
Patient 23	6	47	50	0.32	380	100	0.76
Mean ± SD		64 ± 50*	34 ± 25*	0.34 ± 0.10*	354 ± 77	100 ± 0	0.85 ± 0.11

*Notice the significant decrease of mean peak saccade velocity and HIMP HVOR gain on their lesioned sides compared with those in their intact sides. In addition, the percentage of saccade response is decreased on head turns toward the lesioned side (34%) compared with the one obtained in head turns on the intact side (100%)*.

*indicates significantly difference in t-test, p < 0.05.

#### Chronic UVL Patients

Chronic UVL patients were separated in 3 subgroups based on their pathologies: 7 patients operated with vestibular schwannoma, 8 patients treated with gamma-knife therapy because of unilateral vestibular schwannoma, or 13 patients suffering from Meniere’s disease and treated by intratympanic gentamycin injections (three IT gentamycin injections at 1-week interval). Most operated patients showed very low peak saccade velocity and very low HVOR gain to HIMPs (Figure 4B). The peak saccade velocity in gamma knife-treated patients varies ranging from 50 to 414°/s, and the VOR gain ranging from 0.24 to 0.77. Patients with Meniere’s disease and treated by intratympanic gentamycin injections mostly had peak saccade velocity from 165 to 320°/s, and VOR gain from 0.31 to 0.69. The ratio of the peak saccade velocity to peak head velocity to the lesioned side of chronic UVL group was from 0 to 2.08, indicating different levels of severity of horizontal canal loss of chronic patients (Figure 5B). Despite the pathological diversity in chronic patients, the mean peak saccade velocity in the lesioned side (202 ± 129°/s) of all chronic UVL patients was significantly lower compared with that in their intact side (329 ± 86°/s) (Table 1). The lesioned side of chronic UVL patients produced saccades with a mean latency of 235 ± 48 ms, which was significantly higher compared with that in normal subjects (201 ± 32 ms) (*p* < 0.02).

### Bilateral Vestibular Loss Patients

Few induced saccades in SHIMPs were detected in BVL patient on either side, and the size of the saccades in those few trials was much smaller compared with those of normal subjects (Figure 3C). The peak saccade velocity in SHIMPs (left: 104 ± 81°/s; right: 60 ± 35°/s) and VOR gain (left: 0.25 ± 0.12; right: 0.22 ± 0.17) in HIMPs was dramatically decreased on both sides of BVL patients (Figure 4C, Table 3). The peak saccade velocities on both sides of BVL patients are also not significantly different. The ratio of the peak saccade velocity to the peak head velocity on both sides of BVL patients was low (from 0.05 to 1.37) compared with that in normal subjects (Figure 5C). Also the percentage of saccadic response in BVL patients was significantly lower compared with that in normal subjects with the left side at 36 ± 27% and the right side at 25 ± 14% (Table 3). The latency of saccades for both sides of BVL patients (269 ± 24 ms) was significantly longer compared with that in normal subjects (*p* < 0.0001) (Figure 6).

**Table 3:** **Peak saccade velocity, percentage of response in SHIMPs, and HIMP VOR gain in both sides of BVL patients and normal subjects**.

		Left side			Right side		
		Peak saccade velocity (°/s)	% of response in SHIMP	HVOR gain HIMP	Peak saccade velocity (°/s)	% of response in SHIMP	HVOR gain HIMP
BVL	Patient 1	180	50	0.38	73	27	0.32
	Patient 2	13	5	0.03	10	5	0.01
	Patient 3	175	9	0.21	55	25	0.17
	Patient 4	173	23	0.27	105	45	0.09
	Patient 5	64	64	0.30	31	14	0.20
	Patient 6	20	65	0.28	86	35	0.50

	Mean ± SD	104 ± 81*	36 ± 27*	0.25 ± 0.12*	60 ± 35*	25 ± 14*	0.22 ± 0.17*
Normal	Mean ± SD	347 ± 66	100 ± 0	0.89 ± 0.08	346 ± 61	100 ± 0	1.03 ± 0.10

*Notice the significantly lower anti-compensatory peak saccade velocity and percentage of response in SHIMPs and significantly lower HIMP HVOR gain on both sides of BVL patients compared with the corresponding sides in normal subjects*.

*indicates significantly difference in t-test, p < 0.05.

### DHI and Oscillopsia

No correlation was found between peak saccade velocity in SHIMPs and DHI score in normal subjects, chronic UVL patients, and BVL patients. It agreed with a recent conclusion that DHI had no correlation with vestibular dysfunction in patients tested by HIMP (17). However, all acute UVL patients with small anti-compensatory saccades in SHIMPs complained on oscillopsia in daily life.

### Diagnostic Accuracy

**Figure 7:**
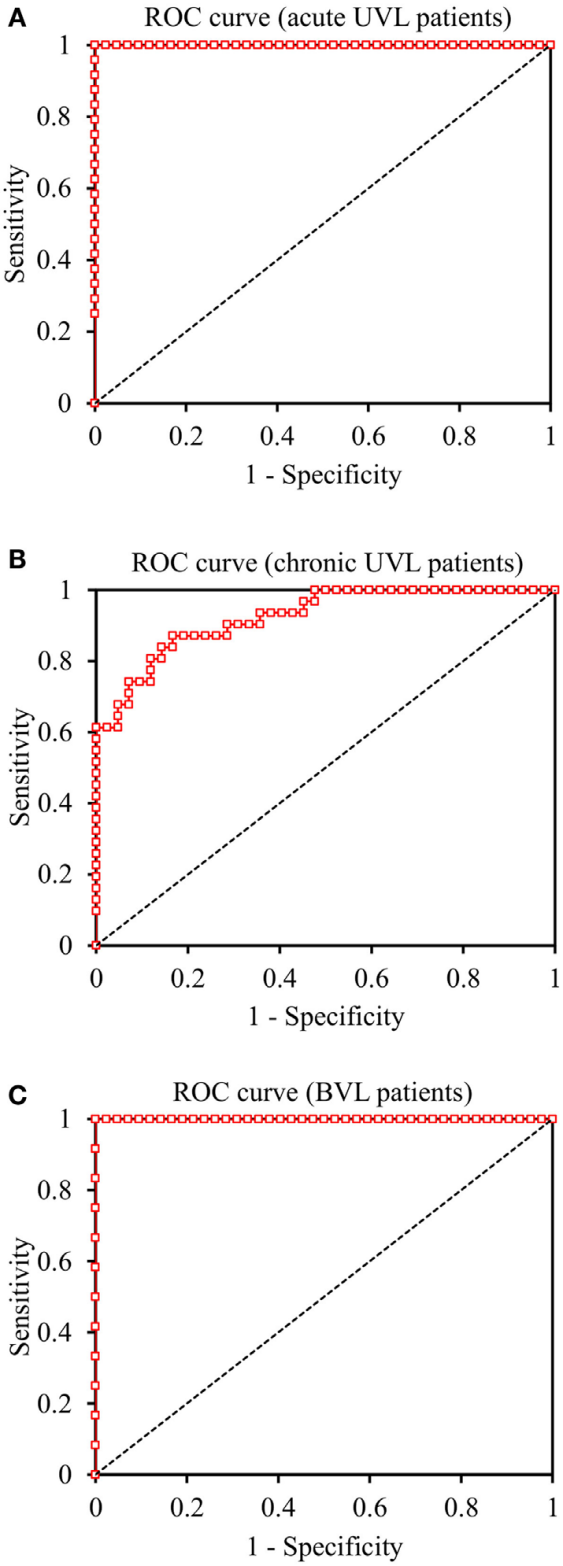
**Receiver Operating Characteristic (ROC) curves of SHIMP test in the discrimination of acute UVL, chronic UVL, and BVL patients**. **(A)** The ratio between mean peak saccade velocity and mean head-peak velocity in SHIMPs with 1.10 discriminated acute UVL patients from healthy controls with 100% sensitivity and 100% specificity. **(B)** The ratio with 1.74 discriminated chronic UVL patients from normal subjects with 87% sensitivity and 83% specificity. **(C)** The ratio with 1.37 discriminated BVL patients from healthy controls with 100% sensitivity and 100% specificity.

The ratio between mean peak saccade velocity and mean head-peak velocity in SHIMPs with 1.10 discriminated acute UVL patients from healthy controls with 100% sensitivity (83–100 95% CI) and 100% specificity (90–100) and an area (AUC) under the ROC curve of 1.0 (1.0–1.0). The ratio between mean peak saccade velocity and mean head-peak velocity with 1.74 discriminated chronic UVL patients from healthy controls with 87% sensitivity (70–95) and 83% specificity (69–92) and an AUC of 0.92 (0.87–0.97). The ratio between mean peak saccade velocity and mean head-peak velocity with 1.37 discriminated BVL patients from healthy controls with 100% sensitivity (71–100) and 100% specificity (90–100) and an AUC of 1.0 (1.0–1.0) (Figure 7).

## Discussion

### The Oculomotor Events during SHIMPs

SHIMP is a simple and easy test with high sensitivity and specificity. In our hands, the instructions were easy to understand and independent of age, the condition, and the social background of the patients. The head impulses needed to be above 130°/s to reveal a significant asymmetry in the vestibular system. We did not observe anticipatory saccades, contrary to what happened with covert saccades during v-HIT. There was also no habituation during the 10 consecutive trials when the clinician encouraged the patient following the target at each trial. Altogether, then, SHIMPs was easy to perform (7), which explains why it could be used in vestibular patients as early as 8 days after surgery of an acoustic schwannoma or after complete IT gentamicin deafferentation.

The oculomotor events during SHIMP can be interpreted as follow:

- At the acute stage of a vestibular lesion, when the head was passively turned toward the intact side, the target was lost because the HVOR drove the eyes to the opposite side of the head rotation and, therefore, away from the target. This occurs because the latency of VOR suppression is around 80 ms (18). Hence, once the passive high velocity, high acceleration rotation of the head had finished, the control subject had to generate a large anti-compensatory saccade reacquire the target, which was aligned with the head. In contrast, when the head was passively turned toward the lesioned side, the HVOR was absent. Therefore, no slow phase could be generated. The eye remained fixed with respect to the head and to the target. Consequently, no saccade was required to catch up the target at the end of the head impulse. More importantly, physicians will be able to assess the vestibular function in acute UVL patients only by performing a few trials in SHIMPs, in which the peak velocity of anti-compensatory saccades successfully indicate the residual function of vestibular system on the lesioned side (Video S1 in Supplementary Material).
- At the chronic stage of a vestibular lesion, vestibular compensation occurred. That is, when the head was turned toward the lesioned side, a HVOR of various gain reappeared (i.e., in patients with Meniere’s disease and treated by intratympanic gentamycin injections) (19). Therefore, the slow phase, it generated, brought the line of sight at distance from the target, which triggered newly formed catch-up saccades. However, in spite of the compensation process, the HVOR gain remained weaker than in control. Hence, the size of the saccades required to catch up the target at the end of the head impulse was most of the time smaller than in control.
- In BVL patients, small saccades in SHIMPs could be observed when the residual function was detected by HIMPs (20).

Altogether, then, SHIMPs presented three interesting features. First, it was easy to perform. Second, as in v-HIT, it allows the evaluation of HVOR gain by measuring the eye and head slow phases at the onset of the head impulse (not performed in this study because we used v-HIT to calculate the HVOR gain). Third, using SHIMPs, we could investigate the capability of a vestibular patient to generate anti-compensatory saccades to acquire a visual target during gaze orientation, despite a deficient HVOR. As explained below, it turned out to be an important point.

Three parameters of the saccades were studied: saccades velocity, the ratio between eye and head velocity, and percentage of saccadic response in SHIMPs. The saccade velocity was a good parameter to compare the capability of the patients to generate anti-compensatory saccades during rotation toward the intact and lesioned side in acute patients. The ratio between eye and head velocity was useful to eliminate false negative due to a too small velocity head turn and to detect a potential vestibular asymmetry at the chronic stage. The percentage of saccadic response can also be a potential indicator of vestibular function in acute UVL and BVL patients as illustrated in Tables 1 and 2. Percentage of response in SHIMPs was 100% when head turn toward the intact side, which is contrast to the percentage of response for head turn toward the lesioned side. Though the number of recruited BVL patients was limited, the data obtained from all patients in BVL group was consistent (Table 3).

As a SHIMP saccade, we take only the anti-compensatory saccades occurring in the first 200 ms following the end of the passive head turn toward the lesion side (i.e., 190 + 200 = 390 ms after the start of the head movement), since the saccades occurring later were of different nature. They occurred when the clinician turned the head back, toward the intact side, to regain the control location. During that return phase, vestibular patients (as the control subjects) performed a few anti-compensatory quick phases intermingled with slow phases of the VOR. These quick phases were, therefore, in opposite direction of the anti-compensatory saccades generated during the rotation toward the lesion side. We also compared the peak saccade velocity in each group of patients at different ages and found that age was not correlated to the peak saccade velocity in any tested groups.

### SHIMPs and v-HIT Complement Each Other

During v-HIT, patients were asked to follow a red earth-fixed spot on the wall, while the clinician imposed head impulses (21). When vestibular function was intact, the HVOR kept the eyes on target. When the vestibular system was lesioned on one or both sides, the target was lost because (a) the HVOR was not fully operational or not operant at all and (b) the head impulses were fast enough to exclude any compensation of the HVOR deficits with the smooth pursuit system. Hence, the eyes ended with an eccentric position with respect to the target and the patients had to generate large compensatory overt and covert saccades to catch up the target during and/or following the head impulse. It was these compensatory catch-up saccades, which allowed detecting the HVOR deficit. Note that for slower passive head rotations, which were not tested here, several small compensatory step saccades can occur. They compensate for the deficient slow phases, i.e., the name of saccadic substitution.

With these characteristics in mind, how is SHIMPs complementary to v-HIT?

First, during v-HIT, the residual slow phases in patients are interrupted by overt saccades. That is measuring the HVOR gain often required removing these catch-up saccades to calculate the ratio between the area under eye velocity and head velocity. It can be difficult in some patients. That never happened for SHIMPs because the residual slow phase always took place before any anti-compensatory saccades could occur, at the end of the head impulses.

Second, in our hands, about 15% of the patients could not be tested easily with v-HIT because they turned the trunk at the same time as the head, or because they had difficulties to focus on the fixed target. Fixating the gaze on a moving target, as it occurred during SHIMPs, turned out to be an easier task in these patients.

**Figure 8:**
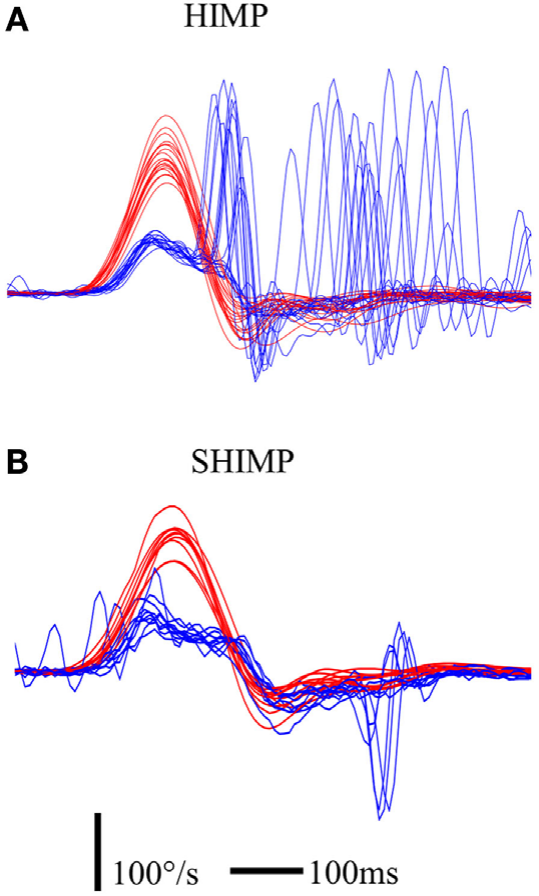
**Illustration of saccadic pattern in HIMP (A) and SHIMP (B) paradigms in a UVL patient suffering from Meniere’s disease and treated by intratympanic gentamycin injections at early stage (3 days after injection)**. The UVL patient was able to make covert compensatory saccades in HIMPs, but not anti-compensatory saccades in SHIMPs. Notice that the HIMP HVOR gain of this patient was 0.41. Red curve: head velocity; blue curve: eye velocity; blue circle: peak saccade velocity; vertical bar: 100°/s; horizontal bar: 100 ms.

Third, as described earlier, during v-HIT, large compensatory saccades always occurred in the patients we tested, and their sizes were indicative of the HVOR deficit. In contrast, for reasons that remain to be elucidated, their capability to generate anti-compensatory saccades during SHIMPs was very variable and did not reflect the gain of their residual HVOR (Figure 8). Very interestingly also, the capability to generate anti-compensatory saccades in a given patient paralleled his/her complaint. The better he/she was at performing SHIMPs saccade, the less he/she complained of his vestibular deficit. Adding these two facts together suggests that training patients to perform anti-compensatory saccades during SHIMPs, when they have difficulties to generate them, could possibly improve their functional outcome and decrease their complaints. A study is under way to test that hypothesis.

## Conclusion

Our study showed that SHIMPs provided important information on vestibular function. The ratio between mean peak saccade velocity and mean head-peak velocity in SHIMPs discriminated vestibular deficit patients from healthy controls with high sensitivity and specificity. In addition, performing HIMPs and SHIMPs in the same patient revealed that compensatory catch-up saccades always occurred during HIMPs, while the anti-compensatory catch-up saccades were more inconsistent during SHIMPs and paralleled the complaints of the patient. It suggested that vestibular information was processed differently to generate these two types of saccade: cortical processing could be more prominent in the case of the SHIMPs anti-compensatory saccades compared with the probably more reflexive covert saccades in v-HIT.

## Author Contributions

CW and IC devised the protocol and wrote much of the paper; QS tested subjects, wrote much of the paper, and conducted the analysis; CM developed the Matlab program for SHIMPs data analysis; GL and OS helped to test patients operated from unilateral vestibular schwannoma; P-PV reviewed the discussion of the paper; and JS participated in statistical analysis.

## Conflict of Interest Statement

The authors declare that the research was conducted in the absence of any commercial or financial relationships that could be construed as a potential conflict of interest.

## Supplementary Material

Video S1 **Stimulation of SHIMPs in a healthy subject**. When head impulses were delivered toward the right side, the healthy subject elicited large anti-compensatory saccades after the head turn. When head impulses were toward the left side, large anti-compensatory saccades also occurred after the head rotation.

Video S2 **Stimulation of SHIMPs in an acute unilateral vestibular loss patient with lesion on the right side**. When head impulses were toward the left intact side, large anti-compensatory saccades occurred after the head rotation. In contrast, when head impulses were delivered toward the right lesioned side, acute UVL patient elicited small anti-compensatory saccades after the head turn.

## Abbreviations

AUC, area under curve; BVL, bilateral vestibular loss; DHI, dizziness handicap inventory; HIMP, conventional head impulse test paradigm; HVOR, horizontal vestibulo–ocular reflex; ROC, receiver operating characteristic; SHIMP, suppression head impulse paradigm; UVL: unilateral vestibular loss; v-HIT, video-head impulse test; VOR, vestibulo–ocular reflex.
